# Pulmonary metastasectomy by VATS or open thoractomy: a systematic review and meta-analysis of long term outcomes

**DOI:** 10.1186/1749-8090-8-S1-O242

**Published:** 2013-09-11

**Authors:** P Herle, M Cheang, P Antippa

**Affiliations:** 1 University of Melbourne, Melbourne, Australia; 2 Royal Melbourne Hospital, Melbourne, Australia

## Background

VATS has become an increasingly popular technique for the cardiothoracic surgeon. Its use in the treatment of malignancy has been an issue of debate previously. Whilst its use has been documented for the treatment of primary lung cancers, its use in metastasectomy has been questioned due to inability to palpate the lung (with the possibility of missing lesions not diagnosed by low sensitivity pre-operative CT) and the association with pleural and port site seeding. There remain no randomized trials and other high level evidence regarding the oncological outcomes of VATS versus open thoracotomy for pulmonary metastases.

## Methods

The study followed the PRISMA protocol for systematic reviews and meta-analyses. The search strategy included an electronic literature review using the PubMed database. The MeSH terms utilized were pulmonary metastasectomy, VATS, thoracoscopic and open. The inclusion criteria for the studies are that they had to have 2 limbs for direct comparison of VATS and open thoracotomies. The studies must also provide data regarding overall survival data or recurrence free survival data separately for the 2 limbs of the study. Meta XL™ and RevMan 5.2 ™ were used for data analysis.

## Results

Nine studies with 796 patients fulfilled the inclusion criteria. The VATS groups had slightly higher odds of 1, 3 and 5 year survival with OR of 1.53, 1.69 and 1.41 respectively. These results demonstrated no heterogeneity on testing. The VATS group also had higher odds of 1, 3 and 5 year recurrence free survival with OR of 1.29, 1.54 and 1.54 respectively for each of these outcomes with no significance in testing for heterogeneity.

Overall pulmonary recurrence had lower odds in the VATS group with an odds ratio of 0.55. This data was also not significantly heterogenous (p = 0.15).

## Conclusions

VATS may present a suitable alternative to open thoracotomy for pulmonary metastasectomy with equivalent survival and recurrence free survival.

